# The association between early country‐level COVID‐19 testing capacity and later COVID‐19 mortality outcomes

**DOI:** 10.1111/irv.12906

**Published:** 2021-10-14

**Authors:** Sneha Kannoth, Sasikiran Kandula, Jeffrey Shaman

**Affiliations:** ^1^ Department of Epidemiology Columbia University Mailman School of Public Health New York New York USA; ^2^ Department of Environmental Health Sciences Columbia University Mailman School of Public Health New York New York USA

**Keywords:** COVID‐19, COVID‐19 mortality, positivity rate, testing capacity

## Abstract

**Background:**

The COVID‐19 pandemic has overrun hospital systems while exacerbating economic hardship and food insecurity on a global scale. In an effort to understand how early action to find and control the virus is associated with cumulative outcomes, we explored how country‐level testing capacity affects later COVID‐19 mortality.

**Methods:**

We used the Our World in Data database to explore testing and mortality records in 27 countries from December 31, 2019, to September 30, 2020; we applied Cox proportional hazards regression to determine the relationship between early COVID‐19 testing capacity (cumulative tests per case) and later COVID‐19 mortality (time to specified mortality thresholds), adjusting for country‐level confounders, including median age, GDP, hospital bed capacity, population density, and nonpharmaceutical interventions.

**Results:**

Higher early testing implementation, as indicated by more cumulative tests per case when mortality was still low, was associated with a lower risk for higher per capita deaths. A sample finding indicated that a higher cumulative number of tests administered per case at the time of six deaths per million persons was associated with a lower risk of reaching 15 deaths per million persons, after adjustment for all confounders (HR = 0.909; *P* = 0.0001).

**Conclusions:**

Countries that developed stronger COVID‐19 testing capacity at early timepoints, as measured by tests administered per case identified, experienced a slower increase of deaths per capita. Thus, this study operationalizes the value of testing and provides empirical evidence that stronger testing capacity at early timepoints is associated with reduced mortality and improved pandemic control.

## 1 INTRODUCTION

As of July 31, 2021, the coronavirus disease 2019 (COVID‐19) pandemic, caused by infection with severe acute respiratory syndrome coronavirus 2 (SARS‐CoV‐2), had produced a total of 197.9 million cases and 4.2 million deaths globally.^1^ Since early 2020, COVID‐19 has inundated healthcare systems throughout the world with increased demand for clinical resources, including intensive care units, hospital beds, and personal protective equipment (PPE).^2^ The public health crisis has additionally worsened mental health outcomes by increasing the prevalence of stress, anxiety, and depression worldwide.^3^, ^4^ Furthermore, COVID‐19 has and will produce adverse socioeconomic outcomes globally as the International Labor Organization projects that the pandemic will push 200 million to unemployment by 2022, and the World Food Program projects that 200 million are at risk of or currently facing acute hunger because of the pandemic.^5^, ^6^

With a continued rise in cases, there is still an essential need to control the spread of SARS‐CoV‐2. To do so, nations initially relied on nonpharmaceutical interventions, including testing, contact tracing, and isolation,^7^ as no universally accepted therapeutic regimen or vaccine was available for this infectious respiratory virus for most of 2020.^8^ Given that the vaccine rollout is currently ongoing throughout the world, measures such as testing, tracing, and isolation still stand as the cornerstones of COVID‐19 control policy.^9^ Nonetheless, over the course of the pandemic, the capacity for recognizing and verifying cases has varied across countries.^9^ Limitations in country‐level testing capacity may stem from shortages in testing equipment, such as swabs, reagents, and PPE, in addition to having a limited number of qualified personnel. Potential limitations in country‐level testing capacity may contribute to increased COVID‐19 case numbers and impact other COVID‐19‐related outcomes.^10^

Currently, few studies have evaluated the effects of early testing capacity strength on future COVID‐19 outcomes.^11^, ^12^ Pan et al. demonstrated that symptom survey rates in Wuhan, China, were indicative of a smaller effective reproductive number of SARS‐CoV‐2 and were associated with reduced daily confirmed cases.^11^ In contrast, Chaudhry et al. adopted a more global approach and found that widespread testing, in addition to full lockdowns, was not associated with country‐level COVID‐19 death rates.^12^ Given such inconsistencies in the literature, we aimed to explore whether early country‐level testing capacity, measured as *tests administered per case identified*, has an association with COVID‐19 mortality. The rationale is that a higher level of testing per case identified is indicative of more proactive efforts to find infections in the broader community and break chains of transmission. We assessed COVID‐19 mortality as opposed to COVID‐19 reported infections, given that testing demand and capacity may change in response to the number of reported infections. In quantifying the effects of testing capacity, researchers may be better able to gauge the value of rapidly scaling up and promoting these public health strategies. Thus, we aimed to assess the relationship between the rigor of early testing and future COVID‐19 mortality across countries.

In exploring this research question, we used information regarding daily cumulative testing and mortality for 27 countries.^1^ We also incorporated data regarding the timing of nonpharmaceutical interventions, including confinement, school/work closures, event cancellations, travel restrictions, and health practices (contact tracing and mask wearing).^13^ We hypothesize that higher country‐level tests per case in the early stages of the outbreak is associated with a lower hazard of reaching higher mortality thresholds (i.e., Y deaths per million), after adjusting for country‐level characteristics, including median age, GDP, hospital bed capacity, population density, and timing of NPI implementation.

## 2 METHODS

### 2.1 Study design and sample

We used the Our World in Data, a publicly available scientific database, to quantify country‐level testing capacity and health outcomes.^1^ The study inclusion criteria broadly identified countries whose first documented testing count was less than 100 tests, and whose case count (at that particular timepoint) was less than the test count. These countries included Bangladesh, Bolivia, Czech Republic, Estonia, Finland, Hungary, Iceland, India, Israel, Italy, Kenya, Latvia, Mexico, Morocco, Nepal, New Zealand, Pakistan, Panama, Paraguay, Poland, Portugal, Serbia, South Africa, South Korea, Switzerland, Tunisia, and the United States (*N* = 27).

### 2.2 Predictor

#### 2.2.1 Early testing capacity

Country‐level testing capacity was operationalized as the number of reported tests per confirmed COVID‐19 case measured at early mortality thresholds, measured at *X* deaths per million, where *X* ranged from 1 to 25.

### 2.3 Outcome

#### 2.3.1 COVID‐19 mortality

COVID‐19 mortality was operationalized as the timespan (measured in days) to reach specific country‐level mortality thresholds, measured at *Y* deaths per million, where *Y* ranged from 2 to 30. The starting point of the timespan was identified as the date corresponding to the early testing capacity measure.

### 2.4 Confounders

In assessing the association between early testing capacity and mortality outcomes, we accounted for potential country‐level confounders, including median age, gross domestic product (GDP), hospital bed capacity (i.e., hospital beds per 1000 people), population density, and nonpharmaceutical intervention (NPI) implementation. NPI categories included (1) mandatory/advised confinement, (2) school/work closures, (3) event cancellations (restrictions on public gatherings, entertainment/cultural sector closures, public services closures), (4) travel restrictions (international travel restrictions, restricted freedom of movement), and (5) health practices (contact tracing, mask wearing). If an NPI category was implemented during the full timespan between a specified date marking early testing capacity and a specified date marking the mortality threshold, then it was assigned a value of 1. The summary NPI variable during this specified time span was expressed as either a sum of the five NPI category values (NPI Sum; range = 0–5) or an average of the five NPI category values (NPI Average; range = 0–1). The summary NPI variable accounted for the time that an NPI was in place and the number of NPIs that were in place. NPI data were retrieved from IBM Research: Worldwide NPI Tracker for COVID‐19.^13^

### 2.5 Statistical analysis

We determined the trajectory of cumulative tests per case and deaths per million for each country in our analysis from December 31, 2019, to September 30, 2020. We additionally modeled the daily time‐evolving pattern of country‐level testing capacity and country‐level mortality rate, in addition to creating a log–log plot assessing the crude relationship between cumulative tests per case and cumulative deaths per million, measured on September 30, 2020, and determined the corresponding Pearson correlation coefficient. We then used Cox proportional hazards regression to assess the association between early testing capacity and later COVID‐19 mortality outcomes. We implemented four models, each adjusting for a different set of covariates. Model 1 adjusted for median age, and GDP at the country‐level; Model 2 adjusted for median age, GDP, and hospital bed capacity at the country‐level; Model 3 adjusted for median age, GDP, hospital bed capacity, and population density at the country‐level; Models 4 and 5 adjusted for median age, GDP, hospital bed capacity, population density, and NPIs, either summed or averaged, at the country‐level. We used R version 4.0.3 to perform all analyses.

### 2.6 Bootstrap analysis

To approximate the variance of the model effect estimates, we conducted 1000 bootstraps on the model coefficients, randomly selecting all 27 countries in the sample, with replacement (Figure S1A–E).

## 3 RESULTS

### 3.1 Global trajectory of testing capacity and COVID‐19 mortality rates

**FIGURE 1:**
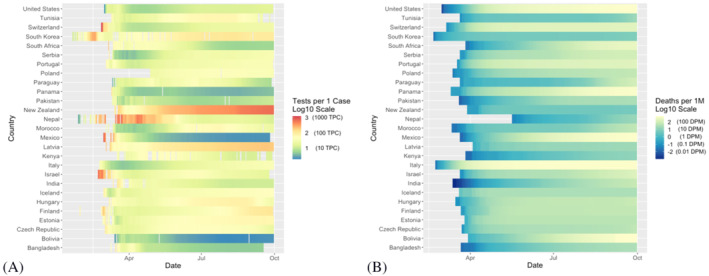
Trajectory of testing capacity and COVID‐19 mortality, across 27 countries (Dec 31, 2019, to Sept 30, 2020). (A) Total tests per case for SARS‐CoV‐2 over time (Log10); (B) Total COVID‐19 deaths per million over time (Log10)

**FIGURE 2:**
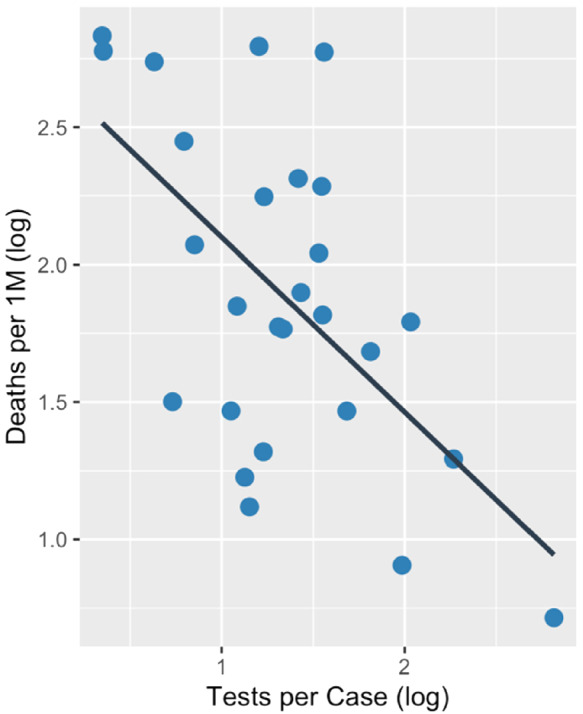
Total COVID‐19 deaths per million vs. total tests per COVID‐19 case on Sept 30, 2020, across 27 countries. Each plot point represents an individual country's total tests per case and total deaths per 1 million. Pearson correlation coefficient: −0.59 (*P* = 0.001)

Figure 1A,B demonstrates the testing capacity and mortality rate trajectory among the 27 specified countries from December 31, 2019, to September 30, 2020. During this time period, we found a crude negative association between log‐transformed tests per case and log‐transformed deaths per million, *r*(25) = −0.59, *P* = 0.001 (Figure 2). Time‐evolving patterns of country‐level testing capacity and country‐level mortality indicate that countries with higher testing capacity early in the pandemic experienced a slower accrual of deaths per capita (Figure S2).

### 3.2 Cox proportional hazards regression analyses

Table 1 provides the adjusted effect estimates regarding the association between tests per case at six deaths per million and time (days) to 15 deaths per million. We found that the number of tests per case at six deaths per million is associated with the time (days) to reach 15 deaths per million, when adjusting for median age and GDP (Model 1: *β* = − 0.057; *P* = 0.003); adjusting for median age, GDP, and hospital bed capacity (Model 2: *β* = − 0.058; *P* = 0.003); adjusting for median age, GDP, hospital bed capacity, and population density (Model 3: *β* = − 0.092; *P* = 0.001); adjusting for median age, GDP, hospital bed capacity, population density, and NPI Sum (Model 4: *β* = − 0.096; *P* = 0.0001); and adjusting for median age, GDP, hospital bed capacity, population density, and NPI Average (Model 5: *β* = − 0.096; *P* = 0.0001). In all instances, the effect estimates indicate that greater tests per case at six deaths per million are positively predictive of a lower risk of reaching 15 deaths per million, that is, a slower accrual of mortality.

**TABLE 1:** Adjusted Cox proportional hazard regression models of tests per COVID‐19 case at six deaths per 1 million and time (days) to 15 COVID‐19 deaths per 1 million

	Estimate	Hazard ratio	SE (estimate)	*P* value
Model 1				
Tests per 1 case at 6 deaths per 1 million	−0.057	0.945	0.019	0.003
Median age	0.014	1.014	0.052	0.797
GDP	0.00007	1.000	0.00003	0.004
Model 2				
Tests per 1 case at 6 deaths per 1 million	−0.058	0.944	0.019	0.003
Median age	−0.012	0.989	0.086	0.894
GDP	0.00008	1.000	0.00003	0.004
Hospital beds per 1000	0.101	1.106	0.273	0.712
Model 3				
Tests per 1 case at 6 deaths per 1 million	−0.092	0.913	0.027	0.001
Median age	0.015	1.015	0.088	0.869
GDP	0.00007	1.000	0.00003	0.008
Hospital beds per 1000	0.015	1.015	0.282	0.957
Population density	−0.002	0.998	0.001	0.036
Model 4				
Tests per 1 case at 6 deaths per 1 million	−0.096	0.909	0.026	0.0001
Median age	0.140	1.150	0.111	0.208
GDP	0.00008	1.000	0.00003	0.004
Hospital beds per 1000	−0.430	0.651	0.376	0.254
Population density	−0.003	0.997	0.001	0.019
NPI^a^ (sum)	1.500	4.480	0.873	0.086
Model 5				
Tests per 1 case at 6 deaths per 1 million	−0.096	0.909	0.026	0.0001
Median age	0.140	1.150	0.111	0.208
GDP	0.00008	1.000	0.00003	0.004
Hospital beds per 1000	−0.430	0.651	0.376	0.254
Population density	−0.003	0.997	0.001	0.019
NPI^a^ (average)	1.500	4.480	0.873	0.086

^a^ Measured country‐level nonpharmaceutical interventions (NPI) in place between 6 deaths per 1 million and 15 deaths per 1 million—NPIs included confinement, school/work closures, event cancellations, travel restrictions, and health practices (mask wearing, contact tracing).

**FIGURE 3:**
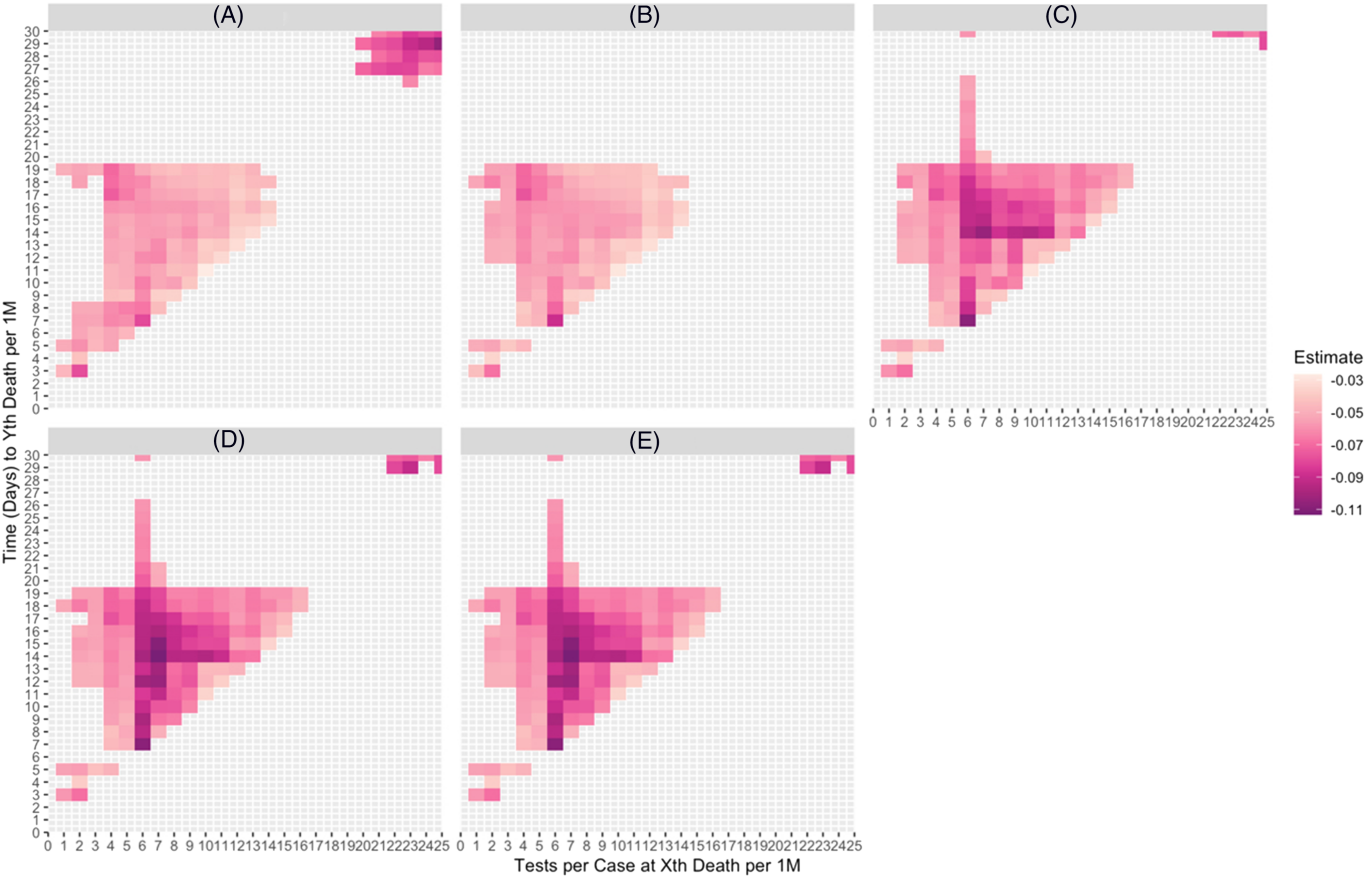
Heatmap plots of Cox proportional hazard regression estimates (for models of tests per COVID‐19 case at *X*th COVID‐19 death per million and time (days) to *Y*th COVID‐19 death per million) across 27 countries. (A) Adjusted for two covariates (median age, GDP). (B) Adjusted for three covariates (median age, GDP, hospital bed capacity). (C) Adjusted for four covariates (median age, GDP, hospital bed capacity, population density). (D) Adjusted for five covariates (median age, GDP, hospital bed capacity, population density, NPI sum). (E) Adjusted for five covariates (median age, GDP, hospital bed capacity, population density, NPI average)

Figure 3A–E indicates heatmap plots corresponding to all effect estimates for Models 1–5, respectively. Shading indicates an association, while varying hues designate the effect size of this association, as noted in the heatmap legend. Across all five models, tests per case at *X* deaths per million (where *X* approximately ranges from 2 to 16) is associated with time to *Y* deaths per million (where *Y* approximately ranges from 7 to 25), after adjusting for the respective model covariates. In all cases, a higher testing capacity at earlier timepoints is indicative of a lower risk of reaching higher mortality thresholds.

## 4 DISCUSSION

We find evidence that early country‐level testing capacity is associated with later country‐level COVID‐19 mortality outcomes, after adjusting for median age, GDP, hospital bed capacity, population density, and NPIs, across 27 countries. More tests per case at an earlier timepoint is associated with a lower risk of reaching higher COVID‐19 mortality levels. Thus, early testing capacity may predict future COVID‐19 mortality outcomes. The findings indicate that early, more aggressive administration of diagnostic testing, as realized in certain countries, may have helped slow the accrual of COVID‐19 deaths.

There is some existing literature that has addressed the mortality impacts of country‐level COVID‐19 testing capacity. Leffler et al. explored the association between viral testing policy and COVID‐19 per‐capita mortality outcomes across 200 countries, up to May 9, 2020. They operationalized country‐level viral testing policy score on the following categorical scale: no implemented policy (0); testing for individuals who are symptomatic with previous exposure (1); testing for individuals who are symptomatic with or without previous exposure (2); or open to all (3). The authors found no association between viral testing policies and COVID‐19 mortality outcomes, using linear regression methods.^14^ Similarly, Chaudhry et al. explored the association between country‐level widespread testing, operationalized as tests per million, and COVID‐19 mortality per capita across 50 countries with the highest case‐load as of April 1, 2020.^12^ They also found no association between country‐level wide‐spread testing and COVID‐19 mortality per capita, using negative binomial regression methods. In contrast, our study, aided in part by an extended study period, finds an association between early country‐level testing capacity and later COVID‐19 mortality outcomes; however, our study differs in that we measure testing capacity as tests administered per case identified. This measure quantifies the number of tests a country is willing to process to find a single case and is thus an estimate of the testing aggressiveness adopted by a country. Thus, our analysis supplements current research in that it demonstrates that there may be a benefit of more abundant testing relative to case numbers.

Here, we use tests per case to estimate the time to particular mortality thresholds. We specifically focus on mortality rates, as tests per case is not an optimal metric for estimating time to cumulative case levels given that more aggressive testing will ascertain a higher fraction of infections as cases. Countries with more active testing strategies may capture a greater number of asymptomatic or paucisymptomatic infections as a result of their more abundant testing and tracing capacity. As such, higher tests per case may slow the accrual of COVID‐19 deaths by supporting more isolation and quarantine measures and thus better inhibiting further transmission of the virus in the community. A robust country‐level testing capacity may also enable earlier diagnosis and treatment of symptomatic cases and improve patient outcomes, thereby also reducing the number of deaths related to COVID‐19.

The major strengths of this study are that we chose to incorporate a time component when measuring mortality outcomes, by operationalizing the outcome as the time interval (in days) to reach a certain country‐level mortality threshold. In using a time to threshold measure, we were able to determine whether stronger testing capacity at the onset of the pandemic may have yielded a sustained impact on later mortality outcomes. Furthermore, we adjusted for potential confounders, such as the timing of NPIs, to assess the public health measures that were in place during a particular period. NPIs encompassed important measures such as masking policies, which have been associated with reduced rates of SARS‐CoV‐2 infection.^15^ We additionally assessed a longer time frame of analysis compared with existing studies, by exploring the association of testing capacity and mortality up until September 30, 2020, utilizing 7 months of longitudinal data.

There were certain limitations in our study design approach. We did not identify the type of diagnostic test used in each country. Countries may have had differential access to rapid antigen tests (RAT), or reverse transcription polymerase chain reaction (RT‐PCR) tests. These differences are important given that RT‐PCR yields higher sensitivity and specificity than RAT, although RAT is relatively quick to administer. We additionally did not account for the type of healthcare system implemented in a country: healthcare access may influence an individual's access to COVID‐19 testing. We also coarsely categorized the NPI variable, which quantified the number of NPIs in place during a particular time period; this variable did not include information regarding the stringency of a specific NPI measure or corresponding public compliance. In addition, we only included countries with daily testing data; thus, the findings may not be applicable to countries that do not report daily testing data. This study also may have yielded limited generalizability, as findings may only correspond to the countries used in this analysis, during this specific period.

Future directions for research may address using a longer time span to determine if this association between early testing capacity and later mortality outcomes continues to hold at longer time intervals. It may also be helpful to explore whether hospitalization rates per capita play a mediating role between country‐level testing capacity and mortality rates in order to assess if lower tests per case rates may increase hospital admissions and overrun the capacity of the healthcare system, which may result in increased COVID‐19 mortality. Future analyses may also consider the effects of new therapeutics and vaccines in preventing COVID‐19 deaths, as this may dilute the association between early testing capacity and later COVID‐19 mortality outcomes.

Overall, this is the first study, to our knowledge, to empirically evaluate the value of a strong country‐level COVID‐19 testing capacity and assess its impact on future COVID‐19 mortality outcomes. This study helps highlight the need in investing in, strengthening, and streamlining COVID‐19 testing strategies so that populations have more feasible access to testing to help mitigate future COVID‐19 outcomes. As countries manage the initial stages of vaccine distribution, investment into COVID‐19 testing should continue to be prioritized. During the early stages of the pandemic in 2020, epidemiologists previously advocated for the rollout of national testing strategies, including centrally located testing facilities, increased access for COVID‐19 test kits, and the mobilization of community resources, such as specialized advisory groups.^16^, ^17^ These approaches should continue to be examined and emphasized to increase the number of administered tests per case identified on a country‐level during the course of the pandemic; this may assist early identification of the SARS‐CoV‐2 infection, prevent further spread of the virus, and help mitigate future COVID‐19 mortality.

## AUTHOR CONTRIBUTIONS

**Sneha Kannoth:** Conceptualization; formal analysis; investigation; methodology; software; validation; visualization. **Sasikiran Kandula:** Conceptualization; formal analysis; investigation; software; validation; visualization. **Jeffrey Shaman:** Conceptualization; formal analysis; methodology; project administration; supervision.

### PEER REVIEW

The peer review history for this article is available at https://publons.com/publon/10.1111/irv.12906.

## Supporting information


**Figure S1** **A–E.** Bootstrap Plots of Cox Proportional Hazard Regression Estimates (For Models of Tests per COVID‐19 Case at X^th^
COVID‐19 Deaths per Million and Time [Days] to Y^th^ COVID‐19 Deaths per Million) Across 27 Countries^a‐e^
^a^Figure Suppl 1A. Adjusted for 2 covariates (Median Age, GDP)
^b^Figure Suppl 1B. Adjusted for 3 covariates (Median Age, GDP, Hospital Bed Capacity)
^c^Figure Suppl 1C. Adjusted for 4 covariates (Median Age, GDP, Hospital Bed Capacity, Population Density)
^d^Figure Suppl 1D Adjusted for 5 covariates (Median Age, GDP, Hospital Bed Capacity, Population Density, NPI Sum)
^e^Figure Suppl 1E. Adjusted for 5 covariates (Median Age, GDP, Hospital Bed Capacity, Population Density, NPI Average)

**Figure S2.** Supporting information

## Data Availability

The data that support the findings of this study are openly available in Our World in Data (url: https://ourworldindata.org/coronavirus) and Worldwide Non‐pharmaceutical Interventions Tracker for COVID‐19 (url: https://ibm.github.io/wntrac/dataset).

## References

[1] [dataset] Roser M , Ritchie H , Ortiz‐Ospina E, Hasell J (2020) ‐ "Coronavirus Pandemic (COVID‐19)". Published online at OurWorldInData.org. Retrieved from: https://ourworldindata.org/coronavirus [Online Resource]

[2] Moghadas SM , Shoukat A , Fitzpatrick MC , et al. Projecting hospital utilization during the COVID‐19 outbreaks in the United States. Proc Natl Acad Sci U S a. 2020 Apr 21;117(16):9122‐9126. 32245814 10.1073/pnas.2004064117 PMC7183199

[3] Salari N , Hosseinian‐Far A , Jalali R , et al. Prevalence of stress, anxiety, depression among the general population during the COVID‐19 pandemic: a systematic review and meta‐analysis. Global Health. 2020 Dec;16(1):57. 32631403 10.1186/s12992-020-00589-w PMC7338126

[4] Ettman CK , Abdalla SM , Cohen GH , Sampson L , Vivier PM , Galea S . Prevalence of depression symptoms in US adults before and during the COVID‐19 pandemic. JAMA Netw Open. 2020 Sep 1;3(9):e2019686. 32876685 10.1001/jamanetworkopen.2020.19686 PMC7489837

[5] Laborde D , Martin W , Vos R . Poverty and food insecurity could grow dramatically as COVID‐19 spreads. Washington, DC: International Food Policy Research Institute (IFPRI); 2020 Apr 16.

[6] Berg J , Hilal A , El S , Horne R . World employment and social outlook: Trends 2021. International Labour Organization; 2021.

[7] Davies NG , Kucharski AJ , Eggo RM , et al. Effects of non‐pharmaceutical interventions on COVID‐19 cases, deaths, and demand for hospital services in the UK: a modelling study. Lancet Public Health. 2020 Jul;5(7):e375‐e385. 32502389 10.1016/S2468-2667(20)30133-X PMC7266572

[8] Jamwal S , Gautam A , Elsworth J , Kumar M , Chawla R , Kumar P . An updated insight into the molecular pathogenesis, secondary complications and potential therapeutics of COVID‐19 pandemic. Life Sci. 2020 Sep 17;257:118105. 32687917 10.1016/j.lfs.2020.118105 PMC7366108

[9] Kandel N , Chungong S , Omaar A , Xing J . Health security capacities in the context of COVID‐19 outbreak: an analysis of International Health Regulations annual report data from 182 countries. Lancet. 2020 Mar 28;395(10229):1047‐1053. 32199075 10.1016/S0140-6736(20)30553-5 PMC7271261

[10] Pettit SD , Jerome KR , Rouquié D , et al. ‘All In’: A pragmatic framework for COVID‐19 testing and action on a global scale. EMBO Mol Med. 2020 Jun 8;12:e12634. 32375201 10.15252/emmm.202012634 PMC7267598

[11] Pan A , Liu L , Wang C , et al. Association of public health interventions with the epidemiology of the COVID‐19 outbreak in Wuhan. China JAMA. 2020 May 19;323(19):1915‐1923. 32275295 10.1001/jama.2020.6130 PMC7149375

[12] Chaudhry R , Dranitsaris G , Mubashir T , Bartoszko J , Riazi S . A country level analysis measuring the impact of government actions, country preparedness and socioeconomic factors on COVID‐19 mortality and related health outcomes. EClinicalMedicine. 2020 Aug 1;25:100464. 32838237 10.1016/j.eclinm.2020.100464 PMC7372278

[13] [dataset] Suryanarayanan P , Tsou CH, & Poddar A, et al. WNTRAC: Artificial Intelligence Assisted Tracking of Non‐pharmaceutical Interventions Implemented Worldwide for COVID‐19. arXiv preprint arXiv:2009.07057. 2020 Sep 2.

[14] Leffler CT , Ing EB , Lykins JD , Hogan MC , McKeown CA , Grzybowski A . Association of country‐wide coronavirus mortality with demographics, testing, lockdowns, and public wearing of masks. Am J Trop Med Hyg. 2020 Dec;103(6):2400‐2411. 33124541 10.4269/ajtmh.20-1015 PMC7695060

[15] Wang X , Ferro EG , Zhou G , Hashimoto D , Bhatt DL . Association between universal masking in a health care system and SARS‐CoV‐2 positivity among health care workers. Jama. 2020 Jul 14;324(7):703‐704. 32663246 10.1001/jama.2020.12897 PMC7362190

[16] Peto J . COVID‐19 mass testing facilities could end the epidemic rapidly. Bmj. 2020 Mar 22;368:m1163. 32201376 10.1136/bmj.m1163

[17] Peto J , Alwan NA , Godfrey KM , et al. Universal weekly testing as the UK COVID‐19 lockdown exit strategy. Lancet. 2020 May 2;395(10234):1420‐1421. 32325027 10.1016/S0140-6736(20)30936-3 PMC7172826

