# Supplementary figures and images for: The association between early country‐level COVID‐19 testing capacity and later COVID‐19 mortality outcomes
Source: Influenza Other Respir Viruses. 2021 Oct 14;16(1):56–62. doi: 10.1111/irv.12906 (PMC8652724; doi:10.1111/irv.12906)

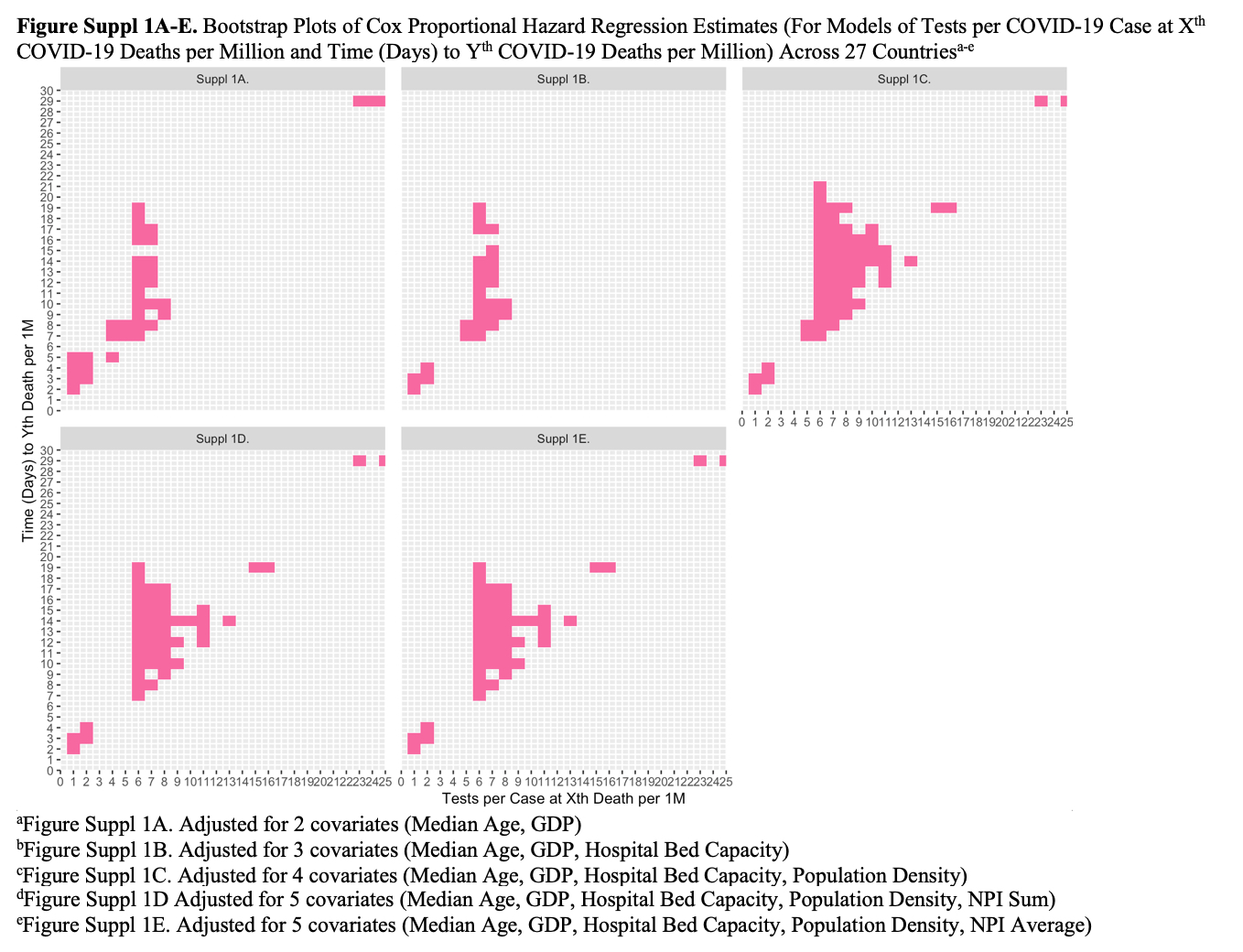

Supplement: Supplementary file 1 — Figure S1 A–E. Bootstrap Plots of Cox Proportional Hazard Regression Estimates (For Models of Tests per COVID‐19 Case at Xth COVID‐19 Deaths per Million and Time [Days] to Yth COVID‐19 Deaths per Million) Across 27 Countriesa‐e aFigure Suppl 1A. Adjusted for 2 covariates (Median Age, GDP) bFigure Suppl 1B. Adjusted for 3 covariates (Median Age, GDP, Hospital Bed Capacity) cFigure Suppl 1C. Adjusted for 4 covariates (Median Age, GDP, Hospital Bed Capacity, Population Density) dFigure Suppl 1D Adjusted for 5 covariates (Median Age, GDP, Hospital Bed Capacity, Population Density, NPI Sum) eFigure Suppl 1E. Adjusted for 5 covariates (Median Age, GDP, Hospital Bed Capacity, Population Density, NPI Average) [file IRV-16-56-s002.jpg]
